# Dysregulation of innate cell types in the hepatic immune microenvironment of alcoholic liver cirrhosis

**DOI:** 10.3389/fimmu.2023.1034356

**Published:** 2023-02-09

**Authors:** Ao Ren, Wenjing He, Jiawei Rao, Dongmei Ye, Pengrui Cheng, Qian Jian, Zongli Fu, Xuzhi Zhang, Ronghai Deng, Yifang Gao, Yi Ma

**Affiliations:** ^1^ Organ Transplant Center, The First Affiliated Hospital, Sun Yat-sen University, Guangzhou, China; ^2^ Guangdong Provincial Key Laboratory of Organ Donation and Transplant Immunology, The First Affiliated Hospital, Sun Yat-sen University, Guangzhou, China; ^3^ Guangdong Provincial International Cooperation Base of Science and Technology (Organ Transplantation), The First Affiliated Hospital, Sun Yat-sen University, Guangzhou, China

**Keywords:** scRNA-seq, MAIT cells, NKT cells, macrophages, fibrosis

## Abstract

**Introduction:**

The risk of alcoholic cirrhosis increases in a dose- and time-dependent manner with alcohol consumption and ethanol metabolism in the liver. Currently, no effective antifibrotic therapies are available. We aimed to obtain a better understanding of the cellular and molecular mechanisms involved in the pathogenesis of liver cirrhosis.

**Methods:**

We performed single-cell RNA-sequencing to analyze immune cells from the liver tissue and peripheral blood form patients with alcoholic cirrhosis and healthy controls to profile the transcriptomes of more than 100,000 single human cells and yield molecular definitions for non-parenchymal cell types. In addition, we performed single-cell RNA-sequencing analysis to reveal the immune microenvironment related to alcoholic liver cirrhosis. Hematoxylin and eosin, Immunofluorescence staining and Flow cytometric analysis were employed to study the difference between tissues and cells with or without alcoholic cirrhosis.

**Results:**

We identified a fibrosis-associated M1 subpopulation of macrophages that expands in liver fibrosis, differentiates from circulating monocytes, and is pro-fibrogenic. We also define mucosal-associated invariant T (MAIT) cells that expand in alcoholic cirrhosis and are topographically restricted to the fibrotic niche. Multilineage modeling of ligand and receptor interactions between the fibrosis-associated macrophages, MAIT, and NK cells revealed the intra-fibrotic activity of several pro-fibrogenic pathways, including responses to cytokines and antigen processing and presentation, natural killer cell-mediated cytotoxicity, cell adhesion molecules, Th1/Th2/Th17 cell differentiation, IL-17 signaling pathway, and Toll-like receptor signaling pathway.

**Discussion:**

Our work dissects unanticipated aspects of the cellular and molecular basis of human organ alcoholic fibrosis at the single-cell level and provides a conceptual framework for the discovery of rational therapeutic targets in liver alcoholic cirrhosis.

## Introduction

Liver cirrhosis is a morbid, multisystem disease associated with frequent hospitalizations and high mortality rates, accounting for 1.2 million annual deaths worldwide and ranking as the 14^th^ and 10^th^ leading cause of death in the world and most developed countries, respectively (1–3). The risk of alcoholic cirrhosis increases in a dose- and time-dependent manner with alcohol consumption, and is related to one’s ethanol metabolism ability (4, 5). With continued alcohol consumption, the alcoholic liver disease progresses to a stage of severely damaged liver cells known as “alcoholic cirrhosis”, which is characterized by progressive hepatic fibrosis and nodules (6). Hence, effective antifibrotic therapies are urgently needed for patients with alcoholic liver cirrhosis (7).

The liver hosts a complex range of immunocompetent cells under physiological and pathological conditions, which may interact with other non-parenchymal cell (NPC) lineages, such as endothelial and mesenchymal cells, to promote the progression of liver fibrosis (8, 9). Gao et al. reviewed inflammatory pathways in alcoholic steatohepatitis and showed that natural killer (NK) T cells may promote alcoholic liver injury *via* the activation of macrophages and that mucosa-associated invariant T cells (MAIT) cells are reduced in patients with alcohol-related liver disease (ALD) (10–12). These immune cells might be associated with the pathogenesis of alcoholic liver cirrhosis and may provide a target for future treatments. Therefore, understanding the functional heterogeneity and interactome of cell lineages that contribute to the fibrotic niche of alcoholic cirrhosis is necessary.

Single-cell RNA sequencing (scRNA-seq) allows us to explore disease mechanisms and individual cell populations at an unprecedented resolution. In this study, using scRNA-seq, we profiled mononuclear immune cells from the liver and blood of patients with alcoholic cirrhosis and healthy patients.

## Methods

Three patients were pathologically diagnosed with alcoholic cirrhosis, and two donors of liver transplantation as healthy controls at The First Affiliated Hospital, Sun Yat sen University. The available clinicopathological features of these patients were summarized in **Supplementary Tables 1**. Admission criteria: onset of jaundice within prior 8weeks; ongoing consumption of>40(female) or 60(male)g alcohol/day for ≥6 months, with<60 days of abstinence before the onset of jaundice; AST>50, AST/ALT>1.5, and both values<400IU/L; serum total bilirubin>3.0 mg/dL; pathological findings confirmed cirrhosis. Exclusion criteria: hepatic failure;hepatitis virus, nonalcoholic fatty and autoimmune liver disease. Informed consent for the sample collection for single-cell RNA sequencing was obtained from the donor’s family delegate. Organs were managed according to organ procurement guidelines. This study was approved by the Ethics Committee for Clinical Research and Animal Trials of the First Affiliated Hospital of Sun Yat-sen University (permit no. [2021]113) and was conducted in accordance with the Declaration of Helsinki principles.

### Hematoxylin and eosin and immunofluorescence analyses

Small pieces of freshly dissected liver were fixed with 10% formalin, embedded in paraffin, cut into 3–5 µm thick sections, and stained with anti-CD80, anti-68, and anti-CD206 monoclonal antibodies (mAbs), according to the manufacturer’s protocols. M1 macrophages were defined as CD68+CD80+, while M2 macrophages were defined as CD68+CD206+.

### Flow cytometric analysis

Liver cells were stained with the indicated fluorescent mAbs and analyzed on a flow cytometer (BD LSRFortessa, USA). MAIT cells were defined as CD3+CD161+TCRVα7.2+. With respect to macrophages analysis, liver cells were stained with the indicated fluorescent conjugated mAbs and analyzed on a full spectrum flow cytometer (Cytek Aurora, USA). M1 macrophages were defined as CD45+CD68+CD86+CD80+, and M2 macrophages were defined as CD45+CD68+CD163+CD206+. Data were analyzed using the FlowJo software (v10.5.3).

### Single-cell RNA statistical analysis

Single-cell RNA (scRNA-seq) data analysis was performed by NovelBio Co., Ltd. with NovelBrain Cloud Analysis Platform (www.novelbrain.com). We applied fastp (13) with default parameters, filtered the adapter sequence, and removed low-quality reads to achieve clean data. Then, the feature-barcode matrices were obtained by aligning reads to the human genome (GRCh38 Ensemble: version 100) using CellRanger v5.0.1. We performed the downsample analysis among samples sequenced according to the mapped barcoded reads per cell of each sample and finally achieved the aggregated matrix. Cells containing over 200 expressed genes and mitochondrial UMI rates below 20% passed the cell quality filtering, and mitochondrial genes were removed from the expression table.

The Seurat package (version: 3.1.4, https://satijalab.org/seurat/) was used for cell normalization and regression based on the expression table, UMI counts of each sample, and percentage of mitochondrial rate to obtain the scaled data. Principal component analysis (PCA) was constructed based on the scaled data with the top 2000 highly variable genes, and the top 10 principals were used for UMAP construction. Using the graph-based cluster method, we acquired the unsupervised cell cluster result based on the PCA top 10 principal, and we calculated the marker genes using the FindAllMarkers function with the Wilcoxon rank-sum test algorithm under the following criteria:1. lnFC > 0.25; 2. P< 0.05; 3. min.pct > 0.1. To identify the cell type in detail, clusters of the same cell type were selected for re-tSNE analysis, graph-based clustering, and marker analysis.

### Pseudotime analysis

Single-cell trajectory analysis was performed using Monocle2 (http://cole-trapnell-lab.github.io/monocle-release) using DDR-Tree and default parameters. Before Monocle analysis, we selected marker genes based on the Seurat clustering results and raw expression counts of the filtered cells. Based on the pseudotime analysis, branch expression analysis modeling (BEAM analysis) was applied for branch fate-determined gene analysis.

### Differential gene expression analysis

To identify differentially expressed genes (DEG) among samples, the function FindMarkers with the Wilcoxon rank-sum test algorithm was used under the following criteria:1. lnFC > 0.25; 2. P< 0.05; 3. min.pct>0.1.

### Cell communication analysis

To enable a systematic analysis of cell-cell communication molecules, we applied cell communication analysis based on the Cell Phone DB toolset (14), a public repository of ligands, receptors, and their interactions. The membrane, secreted, and peripheral proteins of the cluster at different time points were annotated. Significant mean and cell communication significance (P < 0.05) were calculated based on the interaction and the normalized cell-matrix achieved by Seurat normalization.

### Gene enrichment and co-regulated gene analyses

To characterize the relative activation of a given gene set, such as pathway activation, we performed QuSAGE (2.16.1) analysis (15). To characterize the relative activation of a given gene set, such as the KEGG PATHWAY gene set, we performed GSVA (1.32.0) to calculate it for each cell. To discover the gene co-regulation network, the “find gene modules” function of monocle3 was used with the default parameters (16).

### Data-driven regression discontinuity plot

To display the expression levels of chemokine genes, cytokine genes, and anti-tags in cell species, we used the Seurat package (version: 3.1.4, https://satijalab.org/seurat/) to draw feature, bubble, and violin plots.

### Statistical analyses

Statistical analyses of zonation figures were performed using GraphPad Prism v5.0. Data are expressed as mean ± standard deviation, and the statistical test used is listed in each figure caption. Statistical significance was evaluated using ANOVA with a Bonferroni post-test∗∗∗ P < 0.001, ∗∗ P < 0.01, ∗ P < 0.05.

## Results

### A single-cell atlas of human Liver NPCs

**Figure 1:**
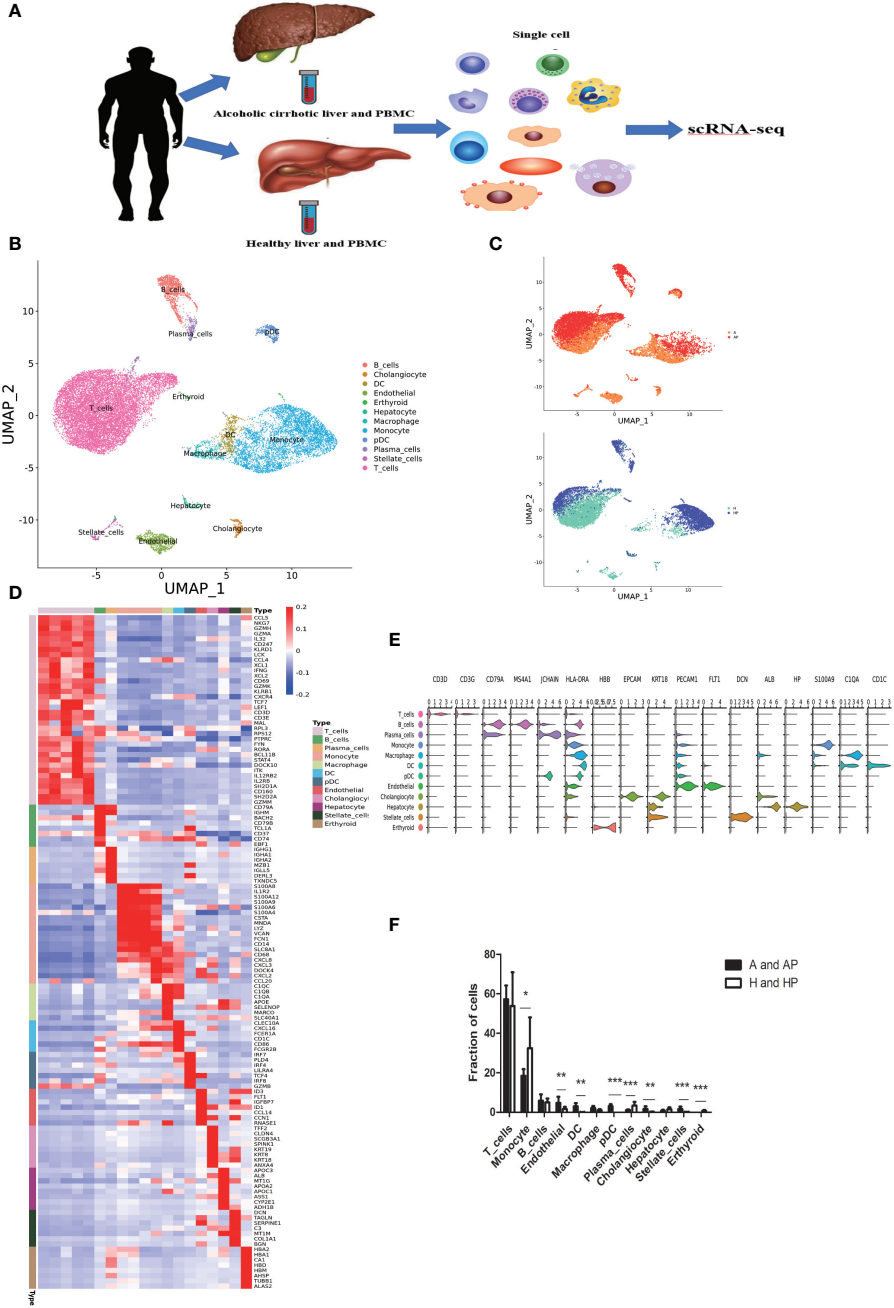
Single-cell atlas of human liver non-parenchymal cells (NPCs). **(A)** An overview of the isolation, FACS, and single-cell sequencing (scRNA-seq) analyses of NPC fractions from liver tissue and peripheral blood mononuclear cells (PBMCs). **(B)** Clustering of 21638 liver-resident cells and PBMCs from five liver tissue and peripheral blood samples (n = 2 healthy and n = 3 alcoholic cirrhosis) **(C)** Annotation of the conditions of the patients. **(D)** Heatmap of cluster marker genes. **(E)** Scaled gene expression of NPCs cells. **(F)** Fraction of cells from NPCs. (*P < 0.05, **P < 0.01, ***P < 0.001).

Non-parenchymal cells (NPCs) were isolated from the livers of patients with alcoholic cirrhosis and healthy controls. CD45^+^ leukocytes and other CD45^-^ NPCs were screened by flow cytometry before scRNA-seq analysis. To better understand the pathogenesis of alcoholic cirrhosis and discriminate between liver-resident and circulating leukocytes, we also collected CD45^+^ peripheral blood mononuclear cells (PBMCs) to perform scRNA-seq analysis (**Figure 1A**). The combined liver NPC and PBMC datasets were partitioned into clusters and annotated using signatures of known lineage markers. The datasets were divided into 19 clusters.

Re-clustering of the 21638 liver-resident cells and PBMCs from five livers and peripheral blood (n = 2 healthy; n = 3 alcoholic cirrhosis) revealed 19 populations, across 12 cell lineages, including T, B, plasma, endothelial, dendritic, erythroid, plasmacytoid dendritic, and stellate cells, as well as macrophages and monocytes (**Figures 1B, C**). And patient demographics and clinical information were shown in **Supplementary Table 1**. Subpopulations and markers were identified across all clusters and lineages, and the top differentially expressed genes (DEGs) for the cellular subclusters are presented in the heatmap (**Figure 1D**). Notably, we identified several distinct types of immune cells: T cells characterized with high expression of the cell markers CD3D and CD3G; monocytes, macrophages, and dendritic cells specifically expressing the cell markers CD1C, C1QA, and S100A9; plasmacytoid dendritic cells highly expressing joining chain of multimeric IgA and IgM (JCHAIN) and major histocompatibility complex class II DR alpha (HLA-DRA); erythroid cells with high beta-globin (HBB) expression; cholangiocytes specifically expressing the markers EPCAM and KRT18; endothelial cells highly expressing fms related receptor tyrosine kinase 1 (FLT1) and platelet endothelial cell adhesion molecule-1 (PECAM1); B cells and plasma cells expressing CD79A and MS4; stellate and smooth muscle cells with high decorin (DCN), actin alpha 2 (ACTA2), and transgelin (TAGLN) expression; and hepatocytes highly expressing albumin (ALB) and haptoglobin (HP). The expression patterns of cluster-specific marker genes in cellular populations are shown in **Figure 1E**. T cells, monocytes, and macrophages accounted for approximately 57%, 18%, and 2%, respectively in the A and AP groups, and approximately 53%, 32%, and 1%, respectively in the H and HP groups (**Figure 1F**) (A: Liver tissue of patients with alcoholic cirrhosis; AP: PBMC of patients with alcoholic cirrhosis; H: Liver tissue of healthy patients; HP: PBMC of healthy patients). Therefore, T cells, macrophages, and monocytes accounted for the largest proportion of immune cells. Additionally, the proportion of T cells was higher in the liver tissues of healthy individuals than in PBMCs, while that in patients with alcoholic cirrhosis was higher in liver tissue. Macrophages were more abundant in the liver tissues than in the PBMCS in both healthy individuals and patients with alcoholic liver cirrhosis (**Supplementary Table 2**). There is no consensus regarding which immune cells are responsible for the progression of alcoholic cirrhosis; we hypothesized that it is caused by the interaction of multiple immune cells. Therefore, we analyzed the effects of related immune cells on the progression of alcoholic liver cirrhosis.

### Role of monocytes and macrophages in alcoholic cirrhosis

**Figure 2:**
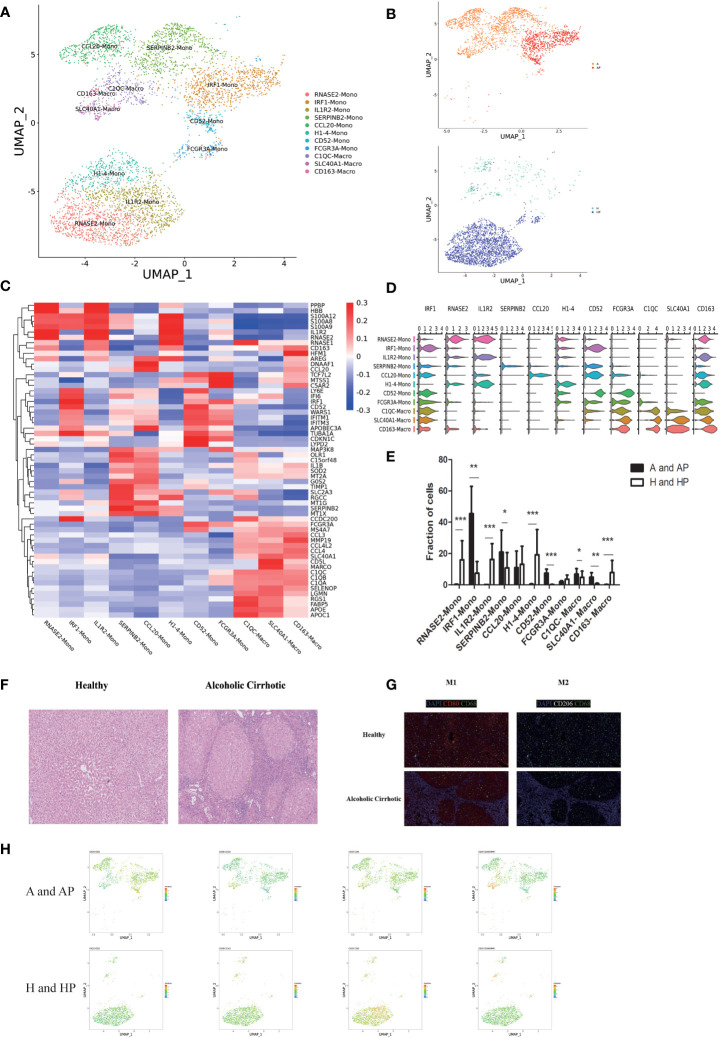
The monocyte and macrophage subpopulations. **(A)** Clustering of 5176 monocytes and macrophages from five liver tissue and peripheral blood samples (n = 2 healthy and n = 3 alcoholic cirrhosis) **(B)** Annotation of monocytes and macrophages by patient’s condition. **(C)** Heatmap of MP cluster marker genes. **(D)** Scaled gene expression of monocytes and macrophages. **(E)** Fraction of monocyte and macrophage cells. **(F)** Pathological changes in the liver tissues of patients with alcoholic cirrhosis (10×). **(G)** Representative immunofluorescent images of CD80 (red), CD206 (white), CD68 (green), and DAPI (blue) in alcoholic cirrhotic livers (10×), and the distribution of M1 and M2 in healthy and alcoholic cirrhotic livers. **(H)** Proteomic analysis of CD80, CD52, CD163, and CD206. (*P < 0.05, **P < 0.01, ***P < 0.001).

Monocytes and macrophages have been shown to play an important role in liver cirrhosis (17). We identified a total of 5176 monocytes and macrophages from five liver and peripheral blood samples (n = 2 healthy and n = 3 alcoholic cirrhosis). Monocytes and macrophages clustered into 11 clusters: IRF1 mono, RNASE2 mono, IL1R2 mono, SERPINB2 mono, CCL20 mono, H1-4 mono, CD52 mono, FCGR3A mono, C1QC macro, SLC40A1 macro, and CD163 macro (**Figures 2A, B**). Subpopulations and markers were identified across all clusters and lineages. The top DEGs for the cellular subclusters are presented in a heatmap in **Figure 2C**, and the expression patterns of the cluster-specific marker genes in the cellular populations are shown in **Figure 2D**. Quantification of monocytes and macrophages revealed that clusters CD52 mono, IRF1 mono, SERPINB2 mono, C1QC macro, and SLC40A1 macro were expanded in liver tissues and PBMCs of patients with alcoholic cirrhotic livers (P < 0.05) (**Figures 2B, E**). In particular, clusters CD52 mono, C1QC macro, and SLC40A1 macro were more enriched in the PBMCs than in the liver tissues of patients with alcoholic liver cirrhosis. SERPINB2 mono was more enriched in the PBMCs than in the liver of patients with alcoholic liver cirrhosis. All the above mentioned clusters were more enriched in patients with alcoholic liver cirrhosis than in healthy individuals. IRF1 mono was more enriched in PBMCs than in the liver tissues of patients with alcoholic liver cirrhosis; however, it was generally more enriched in healthy individuals (**Supplementary Table 3**). Histological analysis confirmed the presence of characteristic pathological changes of cirrhosis, such as destruction of the liver lobule destroyed, fibrous tissue hyperplasia, and pseudolobuli formation, in the liver tissue samples of patients with alcoholic cirrhosis but not in those of healthy individuals (**Figure 2F**). Immunofluorescence analysis showed that M1 macrophages and M2 macrophages (CD206CD68 and CD80CD68, respectively) were more abundant in patients with alcoholic liver cirrhosis than in healthy individuals (**Figure 2G**). Flow cytometric analysis showed that M1 macrophages and M2 macrophages accounted for 54.25 ± 3.35% (mean ± SEM) and 65 ± 6.76% (mean ± SEM) in CD68+macrophages, respectively. (**Supplementary Figure 1**) Furthermore, proteomic analysis revealed that CD80, CD52, CD163, and CD206 were more expressed in the A and AP groups (**Figure 2H**).

**Figure 3:**
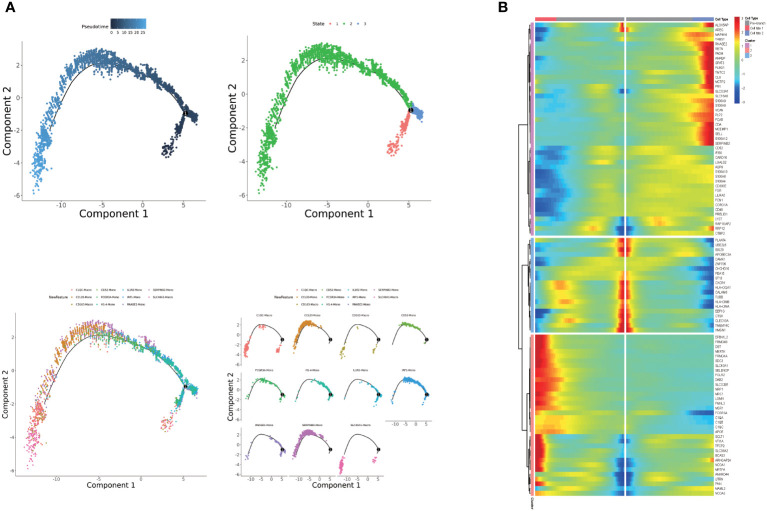
**(A)** Pseudotime analysis of monocyte and macrophage subpopulations. **(B)** Heatmap displaying the expressions of selected marker genes in monocytes and macrophages that are arranged along the pseudotime trajectory.

To determine the origin of macrophages, we performed an *in silico* pseudo-trajectory analysis on a combined dataset of peripheral blood monocytes and liver-resident macrophages (**Figure 3A**). The R package monocle was then used to sort single cells and construct a tree-like structure of the entire lineage differentiation trajectory. Along with trajectory progression, cells experienced three states: the starting point of branching (pre-branch) and the two other branches (Fate1 and Fate2). RNASE2 mono, IRF1 mono, IL1R2 mono, and H1-4 mono were mainly observed at the beginning of the trajectory. CD163 macro, C1QC macro, CCL20 mono, SERPINB2 mono, and SLC40A1 macro were mostly observed at the end of trajectory branch 1. Therefore, our findings suggest that RNASE2 mono, IRF1 mono, IL1R2 mono, and H1-4 mono could be the origin of macrophages and may transform into two distinct types of monocytes and macrophages during the progression of alcoholic cirrhosis. Moreover, the gene expression patterns in the cell state transition along with the progression trajectory were dissected, and 100 dynamic genes with significant expression changes were determined (**Figure 3B**). Our results showed that several crucial drivers, including regulators that are potentially associated with the progression of cirrhosis, such as SLC40A1, DAB2, FOLR2, GPAT3, and CDA, were upregulated during fibrosis. In contrast, transcriptional factors involved in immune cell differentiation and proliferation, such as CD52, CAMK1, HLA-DMB, and CD48, were remarkably downregulated in the trajectory transition process.

### Role of MAIT cells in alcoholic cirrhosis

**Figure 4:**
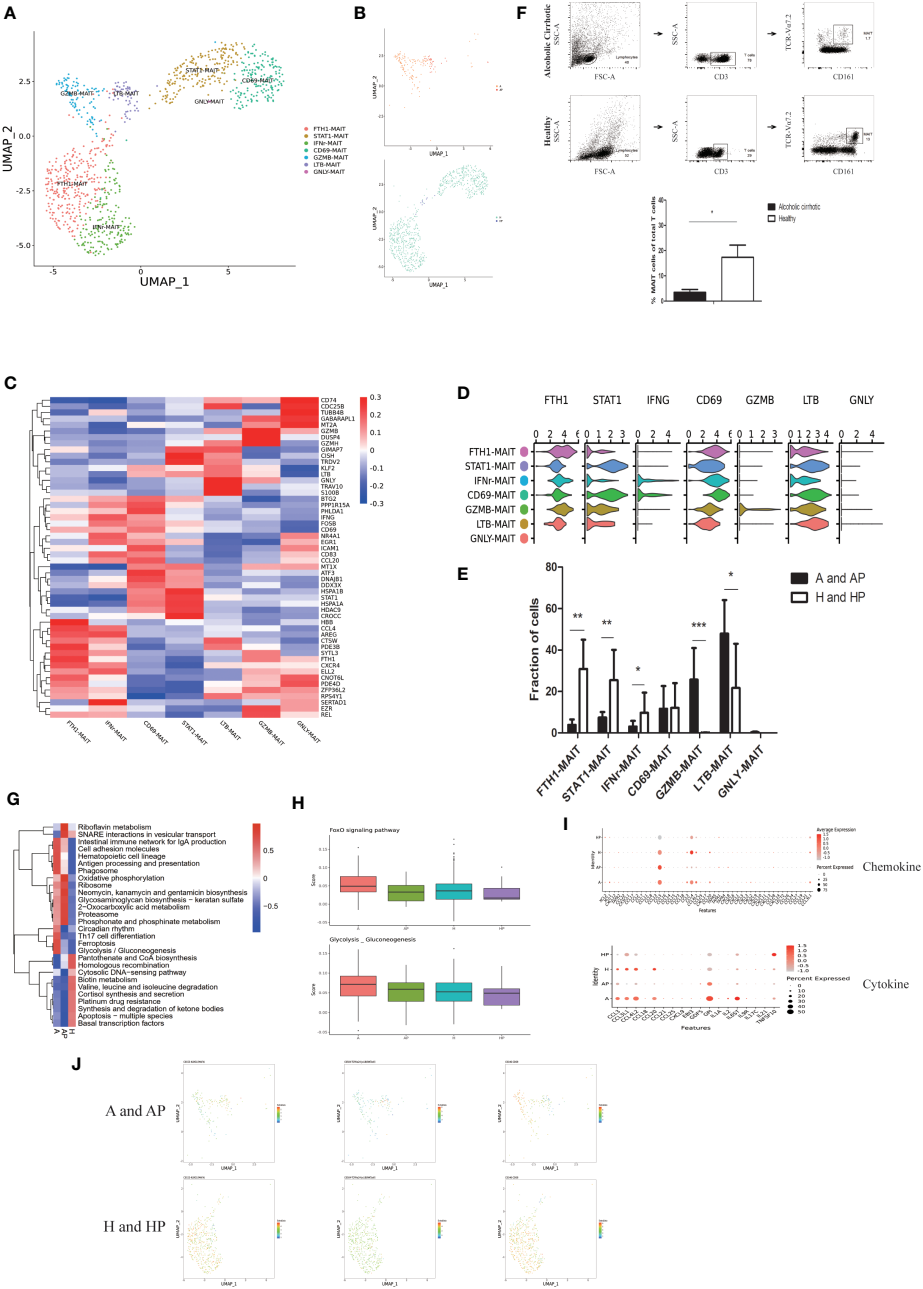
Identifying MAIT cell subpopulations. **(A)** Clustering of 969 mucosal-associated invariant T (MAIT) cells from five liver tissue and peripheral blood samples (n = 2 healthy and n = 3 alcoholic cirrhosis) **(B)** Annotation of MAIT cells by patient’s condition. **(C)** Heatmap of MAIT cell cluster marker genes. **(D)** Scaled gene expression in MAIT cells. **(E)** Fraction of MAIT cells. **(F)** Flow analysis of MAIT cells in each group. **(G)** QuSAGE analysis of MAIT cells in the A, AP, and H groups. **(H)** Single-sample GSEA (ssGSEA) analysis of MAIT cells in the A, AP, H, and HP groups. **(I)** Cytokine and chemokine analyses of MAIT cells in the A, AP, H, and HP groups. **(J)** Proteomic analysis of KLRG1, CD69, and TCR. (*P < 0.05, **P < 0.01, ***P < 0.001).

MAIT cells are present in peripheral blood and are highly abundant in mucosal tissues and the liver. Clustering of human MAIT cells identified seven subpopulations: FTH1-MAIT, STAT1-MAIT, IFNr-MAIT, CD69-MAIT, GZMB-MAIT, LTB-MAIT, and GNLY-MAIT (**Figures 4A, B**). Subpopulations and markers were identified across all clusters and lineages. The DEGs for the cellular subclusters were presented in a heatmap in **Figure 4C**, and the expression patterns of the cluster-specific marker genes in the cellular populations are presented in **Figure 4D**. Quantification of MAIT cells revealed that clusters of GZMB-MAIT cells and LTB-MAIT cells were expanded in the livers and PBMCs of patients with alcoholic cirrhosis (P < 0.05) (**Figures 4B, E**).

GZMB-MAIT cluster was more enriched in the liver than in the PBMCs of patients with alcoholic liver cirrhosis and were more enriched than that in healthy individuals. LTB-MAIT was more enriched in the PBMCs than in the liver of patients with alcoholic liver cirrhosis and less enriched than that in healthy individuals. (**Supplementary Table 4**). Additionally, flow cytometry analysis revealed that MAIT cells were more abundant in healthy individuals than in patients with alcoholic liver cirrhosis (**Figure 4F**).

Furthermore, we performed a quantitative gene set enrichment (QuSAGE) analysis to characterize the relative activation of a given gene set. The patients in the HP group had fewer MAIT cells; thus, they were not included in the analysis. QuSAGE analysis revealed that the most enriched terms in the alcoholic cirrhosis group included antigen processing and presentation, the intestinal immune network for IgA production, glycosaminoglycan biosynthesis, Th17 cell differentiation, ferroptosis, and glycolysis/gluconeogenesis (**Figure 4G**). Moreover, single-sample GSEA (ssGSEA) analysis, an extension of gene set enrichment analysis (GSEA), revealed that the Foxo signaling pathway and glycolysis/gluconeogenesis were significantly upregulated in the A and AP groups (**Figure 4H**). The chemokine analysis, revealed that CCL5, XCL1, CXCL2, CCL2, and CXCL8 were significantly upregulated in the A and AP, groups whereas the cytokine analysis showed that EBI3, GDF5, GPI, IL21, TNFSF10, and IL1A, were significantly upregulated in the A and AP groups (**Figure 4I**). Moreover, the proteomic analysis revealed that CD69 and TCR Vα24-Jα18 were more expressed in the A and AP groups (**Figure 4J**).

### Role of NK cells in alcoholic cirrhosis

**Figure 5:**
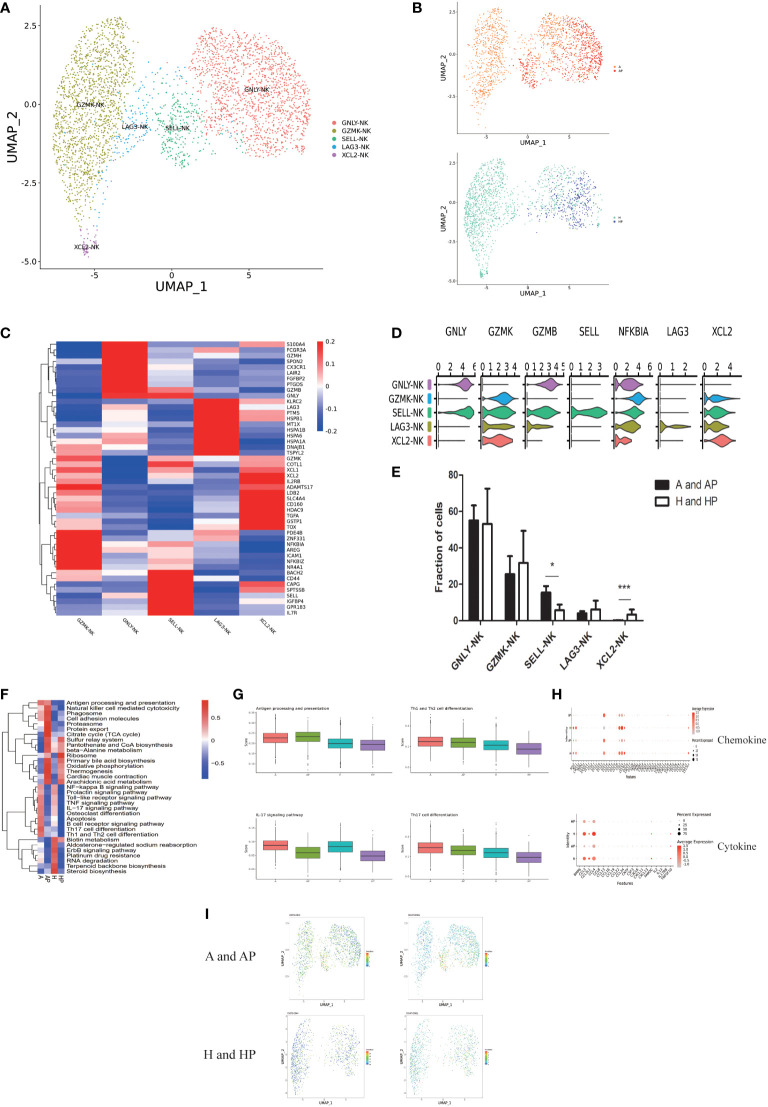
Identifying NK cell subpopulations. **(A)** Clustering of 3578 natural killer (NK) cells from five liver tissue and peripheral blood samples (n = 2 healthy and n = 3 alcoholic cirrhosis). **(B)** Annotation of NK cells by patient’s condition. **(C)** Heatmap of NK cell cluster marker genes. **(D)** Scaled gene expression in NK cells. **(E)** Fraction of NK cells. **(F)** QuSAGE analysis of NK cells in the A, AP, H, and HP groups. **(G)** Single-sample GSEA (ssGSEA) analysis of NK cells in the A, AP, H, and HP groups. **(H)** Cytokine and chemokine analyses of NK cells in the A, AP, H, and HP groups. **(I)** Proteomic analysis of CD62L and CD44. (*P < 0.05, **P < 0.01, ***P < 0.001).

NK cells are thought to be involved in controlling the development and progression of liver fibrosis. Clustering of 3578 NK cells from five livers and peripheral blood (n = 2 healthy and n = 3 alcoholic cirrhosis) was performed and five subpopulations were identified: GNLY NK, GZMK NK, SELL-NK, LAG3 NK, and XCL2 NK (**Figures 5A, B**). Subpopulations and markers were identified across all clusters and lineages. The DEGs for the cellular subclusters are presented in a heatmap in **Figure 5C**, and the expression patterns of the cluster-specific marker genes in the cellular populations are presented in **Figure 5D**. Clusters of SELL-NK were expanded in the livers and PBMCS of patients with alcoholic liver cirrhosis (**Figure 5E**) (P < 0.05) and were more enriched in healthy individuals (**Supplementary Table 5**).

Furthermore, QuSAGE analysis revealed that the most enriched terms in the alcoholic cirrhosis group included antigen processing and presentation, natural killer cell-mediated cytotoxicity, cell adhesion molecules, Th1/Th2/Th17 cell differentiation, IL-17 signaling pathway, and toll-like receptor (TLR) signaling pathway (**Figure 5F**). The HP group was not included in the analysis because they presented a low number of NK cells. Additionally, ssGSEA showed that antigen processing and presentation, IL-17 signaling pathway, and Th1 and Th2 cell differentiation were significantly enhanced in the A and AP groups (**Figure 5G**). In the chemokine analysis, CCL13, CXCL12, CXCL8, CXCL3, and CXCL2 were significantly enhanced in the A and AP groups. The cytokine analysis revealed that BMP6, CCL3, CCL3L1, CNTF, CSF3, CXCL6, IL13, IL17RB, and TNFSF10, among others, were significantly activated in the A and AP groups (**Figure 5H**). The proteomic analysis revealed that CD62L and CD44 were more highly expressed in the A and AP groups (**Figure 5I**).

### Multilineage interactome in the fibrotic niche

**Figure 6:**
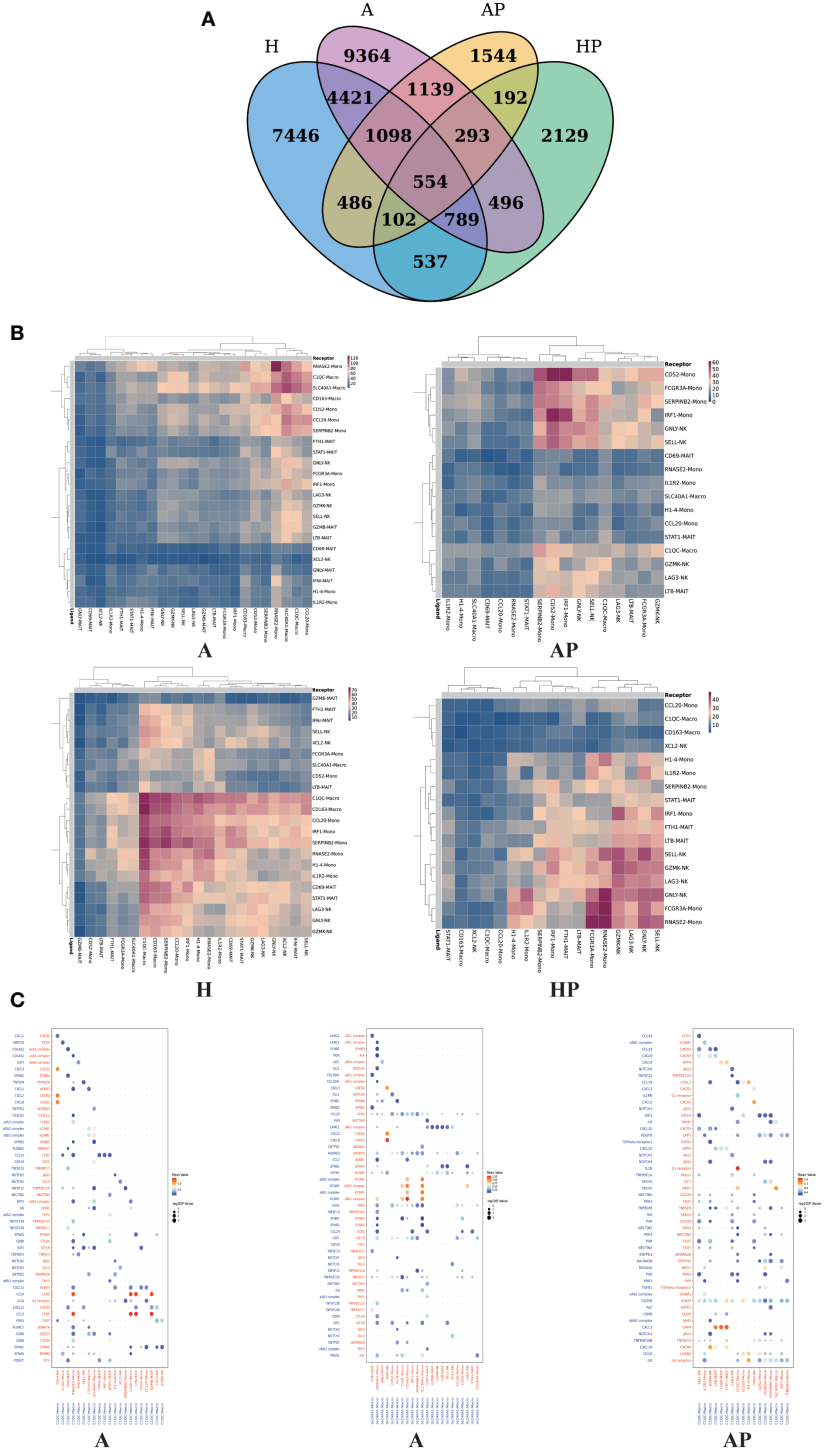
The interactions among NK cells, macrophages, and MAIT cells in the A, AP, H, and HP groups. **(A)** Venn diagram comparing different groups. **(B)** Heatmap showing the interactions among NK cells, macrophages, and MAIT cells in the A, AP, H, and HP groups. **(C)** Dot plot of ligand–receptor interactions among macrophages, MAIT, and NK cells in the A and AP groups.

Having defined the populations of monocytes, macrophages, MAIT cells, and NK cells, we confirmed the close topographical association of these cells with alcoholic cirrhosis. To characterize the immune microenvironment of alcoholic cirrhosis, the Cell Phone toolset was used to detect intercellular communication among immune cells. In the output of the results of the ligand–receptor, the heatmap plot function was used to analyze the interactions among immune cells, which showed that macrophages and monocytes were significantly activated and interacted with MAIT and NK cells (**Figures 6A, B**).

Numerous statistically significant interactions were detected between ligands and cognate receptors expressed by macrophages, MAIT cells, and NK cells. In group A, macrophages, as ligands, interact with NK and MAIT cells. C1QC macro and SLC40A1 as ligands mainly interact with GNLY N, GZMB-MAIT, and LTB-MAIT. In the AP group, the C1QC macro, as a ligand, mainly interacts with GZMK-NK, SELL-NK, GNLY-NK, and LTB-MAIT (**Figure 6B**). In group A, C1QC macro expressed CCL3 and CCL4 signaling to cognate receptors CCR5 on LTB-MAIT and GZMB-MAIT cells, and SLC40A1 macro expressed CXCL8 signaling to cognate receptors CXCR2 on GNLY NK cells. In the AP group, C1QC macro expressed CXCL2 signaling to cognate receptors DPP4 on STAT1 MAIT, CD69 MAIT, and LTB-MAIT cells, and expressed CXCL16 signaling to cognate receptors CXCR6 on GZMK NK cells (**Figure 6C**). Therefore, our unbiased dissection of the key ligand-receptor interactions between fibrosis-associated NPCs highlights macrophages, MAIT, and NK cells as important regulators within the human liver alcoholic fibrotic niche.

## Discussion

The pathogenesis of alcoholic liver cirrhosis remains unknown. Only a few heavy drinkers develop alcoholic cirrhosis, which indicates that there are other factors other than alcohol contributing to the progression of alcoholic cirrhosis (18). Alcoholic cirrhosis is a complex and multifactorial ALD that is affected by the interactions of environmental or host factors, which partly explains the large inter-individual variability in the likelihood of developing cirrhosis and affected by ethanol metabolism.

The deficiency of certain enzymes related to alcohol metabolism could result in alcohol intolerance (18). For instance, there is a clear dose relationship between the amount of alcohol, an environmental factor, and the development of alcoholic cirrhosis (19, 20), whereas coffee seems to protect against ALD (21). Other environmental factors, such as obesity, smoking, and viral hepatitis infections, are recognized as important promoters of ALD (22–25). Several studies have shown that the genetic background, a host factor, might affect the progression of ALD; women are at greater risk of alcohol-related cirrhosis, and race may affect the individual’s alcohol sensitivity, since Hispanic men and women have a higher risk for developing alcoholic cirrhosis than black men and women in the United States (26–28). Additionally, some studies have found that certain variants are associated with the risk of developing ALD, such as PNPLA3, which was found to modulate the evolution of steatosis, necroinflammation, fibrosis, and HCC in alcoholics (29). The pathogenesis of ALD can also be conceptually divided into i) ethanol-mediated liver injury, ii) inflammatory immune response to injury, and iii) intestinal permeability and microbiome changes (30). The inflammatory immune response plays an important role in ALD. The interferon regulatory factor 3 is phosphorylated upon alcohol response, leading to the activation of an inflammatory response, which is required for the intrinsic (mitochondrial) apoptosis pathway in hepatocytes (31, 32). Bacteria-derived LPS interacts with TLR4 of the Kupffer cells, leading to the production of proinflammatory cytokines through the IRF-3 signaling pathway (30). Murine studies identified Interleukin-1 receptor-associated kinase 4 (IRAK4), the master kinase of Toll-like receptor (TLR)/IL-1R-mediated signalling activation, inhibiting IRAK4 kinase activity improves ethanol-induced liver injury in mice (10). Therefore, the immune and inflammatory responses play important roles in the progression of ALD and alcoholic cirrhosis. Here, we explored the relationship between immune cells and alcoholic liver cirrhosis using scRNA-seq.

Inflammatory processes are the primary contributors to the development and progression of ALD (10). A variety of cells in the liver and PBMCs are involved in inflammation, including macrophages, monocytes, NK cells, MAIT cells, which may be contribute to disease severity in patients with severe alcoholic hepatitis (10, 33).. In mild and chronic alcoholic steatohepatitis (ASH), the number of hepatic macrophages is increased, possibly due to infiltrating monocyte-derived macrophages, which also contributes to the pathogenesis of ASH (34). Kupffer cells become sensitized to TLR4-induced signaling after chronic ethanol exposure. These proinflammatory macrophages, together with infiltrating macrophages dictated by LPS, interferon-γ, and granulocyte-macrophage colony stimulatory factor (GM-CSF) signaling, are commonly classified as M1 macrophages as opposed to M2 macrophages that usually arise in Th2 responses in allergy, granuloma formation, and wound healing (10). It is generally believed that activated M1 macrophages produce high amounts of cytokines, such as IL-1β, TNFα, IL-12, IL-18, and IL-23, which help to induce antigen-specific Th1 and Th17 cell inflammatory responses, thereby promoting inflammation. In contrast, activated M2 macrophages secrete large amounts of IL-10, IL-1R antagonist, and TGF-β and subsequently suppress inflammation and promote tissue repair (10). Infiltrating monocytes develop into M1-like hepatic macrophages *via* Notch-1 dependent mitochondrial reprogramming in ASH (35). However, the role of macrophages in the development of alcoholic cirrhosis and the specific underlying mechanisms of the disease remain to be studied.

Patients with ALD have significant infiltration and activation of CD4+ and CD8+ T cells in the liver (36). Studies have shown that NKT cells promote ALD *via* activation of Kupffer cells and macrophages in mice (11). However, the number of NK T cells in humans is low; hence, NKT cells likely play a less important role in the pathogenesis of alcoholic cirrhosis. In contrast, the liver contains MAIT cells, representing 20%–50% of intrahepatic T cells, which play a key role in controlling bacterial infections (10). Patients with ALD exhibit a marked reduction in MAIT cells, possibly contributing to the increased risk of bacterial infections (37). In conclusion, macrophages, NK T cells, and MAIT cells ​may play a role in the development of ALD; however, their role in the progression of alcoholic cirrhosis remains unknown.

Here, using scRNA-seq, we dissected the fibrotic niche of human alcoholic cirrhosis, identifying pathogenic subpopulations of macrophages, NK cells, and MAIT cells producing fibroblasts. Several studies have shown that C1QC and SLC40A1 macrophages are associated with the tumor microenvironment, and patients with high C1QC TAM gene signatures have the best prognosis (38–40). Through Cell Phone analysis, we found that C1QC and SLC40A1 macrophages were also associated with liver fibrosis and cirrhosis, which might act by interacting with NK and MAIT cells. These macrophages might have originated from RNASE2 mono, IRF1 mono, IL1R2 mono, and H1-4 mono. Among CD8+ T lymphocytes, GZMB+ effector cells, which express high levels of cytotoxic molecules, and LTB has been reported to play an important role in fibrosis (41, 42). The SELL gene encodes a cell surface adhesion molecule, L-selectin, which belongs to the adhesion/homing receptor family and plays a role in promoting the migration of leukocytes to lymphoid organs, which plays an important role in the immune response (43, 44).

We dissected a complex, profibrotic interactome among multiple fibrosis-associated cell lineages and identified highly relevant intra-fibrosis pathways that can potentially be therapeutic targets for the treatment of alcoholic liver cirrhosis. In this era of precision medicine, this unbiased multilineage approach can help design highly targeted combination therapies that are necessary for an effective antifibrotic potency. The application of our novel fibrosis-associated cell markers could also potentially inform molecular pathology-based patient stratification, which is fundamental to the prosecution of successful antifibrotic clinical trials. Our study has several limitations: the limited number of samples might bias the results. Also, we confirmed that the interaction between MAIT, NK and macrophage lead to the progression of alcoholic cirrhosis, but its specific mechanism requires further study. Our work illustrates the power of single-cell transcriptomics to determine the cellular and molecular basis of human organ fibrosis, providing a conceptual framework for the discovery of relevant therapeutic targets to treat patients with a broad range of alcoholic fibrotic diseases.

## Data availability statement

The datasets presented in this study can be found in online repositories. The names of the repository/repositories and accession number(s) can be found below: PRJNA928204 (SRA).

## Ethics statement

The studies involving human participants were reviewed and approved by the Ethics Committee for Clinical Research and Animal Trials of the First Affiliated Hospital of Sun Yat-sen University. The patients/participants provided their written informed consent to participate in this study.

## Author contributions

AR, YG and YM designed the study. AR, WH, YG and YM conducted experiments. AR, WH, DY and JR harvested the surgical specimen. AR and YG interpreted the histology. AR, WH, JR, DY, PC, QJ, ZF, RD, YG and YM analyzed and interpreted the data. AR, YG and YM prepared the manuscript with critical revision from all authors. All authors contributed to the article and approved the submitted version. 
