# Supplementary material for: Dysregulation of innate cell types in the hepatic immune microenvironment of alcoholic liver cirrhosis
Source: Front Immunol. 2023 Feb 9;14:1034356. doi: 10.3389/fimmu.2023.1034356 (PMC9947838; doi:10.3389/fimmu.2023.1034356)
Supplement: Supplementary file 1 [file DataSheet_1.docx]

**Supplementary Tables 1**

|  | Alcoholic cirrhosis  (n=3) | Healthy control  (n=2) |
| --- | --- | --- |
| Age(years) | 52.3±5.9 | 19.0±2.8 |
| Gender(Male:Female) | 3:0 | 1:1 |
| MELD Score | 22.0±9.2 | 8.5±3.5 |
| Child-Pugh | 10.3±1.2 | 6.0±1.4 |

**Supplementary Tables 2**

| Type | A | AP | H | HP |
| --- | --- | --- | --- | --- |
| T_cells | 53.92523 | 64.55904 | 83.38672 | 25.47879 |
| Monocyte | 15.58077 | 16.43586 | 4.975443 | 59.17576 |
| B_cells | 1.268358 | 14.23105 | 1.387999 | 8.169697 |
| Endothelial | 8.451268 | 0.091108 | 3.502029 | 0.072727 |
| DC | 5.380507 | 1.184402 | 0.106769 | 0.072727 |
| Macrophage | 4.325768 | 0.255102 | 1.985906 | 0.048485 |
| pDC | 4.445928 | 1.494169 | 0 | 0 |
| Plasma_cells | 1.094793 | 0.911079 | 1.00363 | 4.436364 |
| Cholangiocyte | 2.803738 | 0.054665 | 0.491138 | 0 |
| Hepatocyte | 0.320427 | 0.510204 | 2.754644 | 0.872727 |
| Stellate_cells | 2.363151 | 0.145773 | 0.256246 | 0.048485 |
| Erthyroid | 0.040053 | 0.127551 | 0.149477 | 1.624242 |

A: Liver tissue of patients with alcoholic cirrhosis; AP: PBMC of patients with alcoholic cirrhosis; H: Liver tissue of healthy patients; HP: PBMC of healthy patients

**Supplementary Tables 3**

| Type | A | AP | H | HP |
| --- | --- | --- | --- | --- |
| RNASE2-Mono | 0.650289 | 0.25641 | 2.298851 | 39.74671 |
| IRF1-Mono | 5.274566 | 82.5641 | 17.24138 | 0.292255 |
| IL1R2-Mono | 0.144509 | 0.512821 | 3.065134 | 36.67803 |
| SERPINB2-Mono | 39.16185 | 1.153846 | 24.52107 | 0.097418 |
| CCL20-Mono | 28.17919 | 0.128205 | 22.60536 | 0.048709 |
| H1-4-Mono | 0.433526 | 0.128205 | 3.448276 | 19.48368 |
| CD52-Mono | 2.67341 | 12.69231 | 0.383142 | 0 |
| FCGR3A-Mono | 1.589595 | 1.923077 | 1.149425 | 3.555772 |
| C1QC- Macro | 12.93353 | 0.384615 | 10.72797 | 0.048709 |
| SLC40A1- Macro | 8.526012 | 0.25641 | 1.532567 | 0 |
| CD163- Macro | 0.433526 | 0 | 13.02682 | 0.048709 |

A: Liver tissue of patients with alcoholic cirrhosis; AP: PBMC of patients with alcoholic cirrhosis; H: Liver tissue of healthy patients; HP: PBMC of healthy patients

**Supplementary Tables 4**

| Type |  | A | AP | H | HP |
| --- | --- | --- | --- | --- | --- |
| FTH1-MAIT |  | 9.15493 | 0 | 33.12263 | 22.22222 |
| STAT1-MAIT |  | 3.521127 | 7.407407 | 22.88243 | 11.11111 |
| IFNr-MAIT |  | 2.816901 | 0 | 21.74463 | 0 |
| CD69-MAIT |  | 0.704225 | 7.407407 | 21.36536 | 0 |
| GZMB-MAIT |  | 66.19718 | 0 | 0.252845 | 0 |
| LTB-MAIT |  | 16.19718 | 85.18519 | 0.632111 | 66.66667 |
| GNLY-MAIT |  | 1.408451 | 0 | 0 | 0 |

A: Liver tissue of patients with alcoholic cirrhosis; AP: PBMC of patients with alcoholic cirrhosis; H: Liver tissue of healthy patients; HP: PBMC of healthy patients

**Supplementary Tables 5**

| Type | A | AP | H | HP |
| --- | --- | --- | --- | --- |
| GNLY-NK | 35.65574 | 78.03138 | 21.00076 | 91.46341 |
| GZMK-NK | 51.80328 | 0.570613 | 64.74602 | 1.52439 |
| SELL-NK | 8.114754 | 20.39943 | 2.72934 | 4.878049 |
| LAG3-NK | 4.262295 | 0.998573 | 7.505686 | 1.829268 |
| XCL2-NK | 0.163934 | 0 | 4.018196 | 0.304878 |

A: Liver tissue of patients with alcoholic cirrhosis; AP: PBMC of patients with alcoholic cirrhosis; H: Liver tissue of healthy patients; HP: PBMC of healthy patients

Supplementary Figure 1


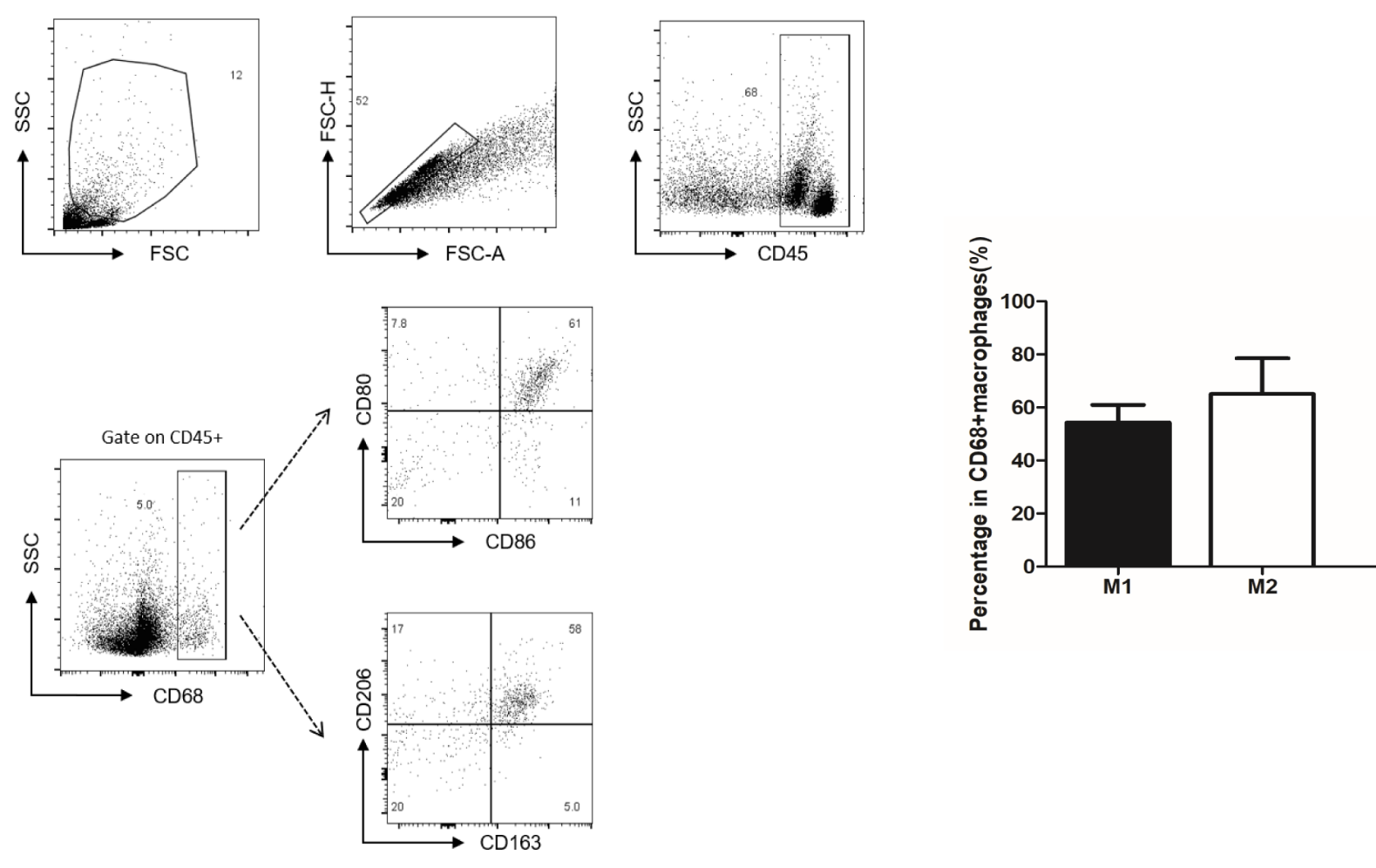


Flow cytometric analysis showed that M1 macrophages and M2 macrophages accounted for 54.25±3.35% (mean±SEM) and 65±6.76% (mean±SEM) in CD68+macrophages in patients with alcoholic liver cirrhosis, respectively(n=4).
